# CRF01_AE-Specific Neutralizing Activity Observed in Plasma Derived from HIV-1-Infected Thai Patients Residing in Northern Thailand: Comparison of Neutralizing Breadth and Potency between Plasma Derived from Rapid and Slow Progressors

**DOI:** 10.1371/journal.pone.0053920

**Published:** 2013-01-07

**Authors:** Sompong Sapsutthipas, Naho Tsuchiya, Panita Pathipavanich, Koya Ariyoshi, Pathom Sawanpanyalert, Naokazu Takeda, Panasda Isarangkura-na-ayuthaya, Masanori Kameoka

**Affiliations:** 1 Thailand-Japan Research Collaboration Center on Emerging and Re-emerging Infections (RCC-ERI), Nonthaburi, Thailand; 2 Institute of Tropical Medicine, Nagasaki University, Nagasaki, Japan; 3 Lampang Hospital, Lampang, Thailand; 4 National Institute of Health, Department of Medical Sciences, Ministry of Public Health, Nonthaburi, Thailand; 5 Research Institute for Microbial Diseases, Osaka University, Osaka, Japan; 6 Department of International Health, Kobe University Graduate School of Health Sciences, Hyogo, Japan; Boston College United States of America

## Abstract

**Background:**

Development of a protective vaccine against human immunodeficiency virus type 1 (HIV-1) is an important subject in the field of medical sciences; however, it has not yet been achieved. Potent and broadly neutralizing antibodies are found in the plasma of some HIV-1-infected patients, whereas such antibody responses have failed to be induced by currently used vaccine antigens. In order to develop effective vaccine antigens, it is important to reveal the molecular mechanism of how strong humoral immune responses are induced in infected patients. As part of such studies, we examined the correlation between the anti-HIV-1 neutralizing antibody response and disease progression.

**Methodology/Principal Findings:**

We evaluated the anti-HIV-1 neutralizing activity of plasma derived from 33 rapid and 34 slow progressors residing in northern Thailand. The level of neutralizing activity varied considerably among plasmas, and no statistically significant differences in the potency and breadth of neutralizing activities were observed overall between plasma derived from rapid and slow progressors; however, plasma of 4 slow progressors showed neutralizing activity against all target viruses, whereas none of the plasma of rapid progressors showed such neutralizing activity. In addition, 21% and 9% of plasmas derived from slow and rapid progressors inhibited the replication of more than 80% of CRF01_AE Env-recombinant viruses tested, respectively. Neutralization of subtype B and C Env-recombinant viruses by the selected plasma was also examined; however, these plasma samples inhibited the replication of only a few viruses tested.

**Conclusions/Significance:**

Although no statistically significant differences were observed in the potency and breadth of anti-HIV-1 neutralizing activities between plasma derived from rapid and slow progressors, several plasma samples derived from slow progressors neutralized CRF01_AE Env-recombinant viruses more frequently than those from rapid progressors. In addition, plasma derived from HIV-1-infected Thai patients showed CRF01_AE-specific neutralizing activity.

## Introduction

More than 30 million individuals are infected with human immunodeficiency virus type 1 (HIV-1) worldwide, and 2.5 million new infections have been estimated to occur yearly; therefore, an HIV-1 vaccine is urgently required. Neutralizing antibodies are a critical component of the protective immunity required for developing an effective HIV-1 vaccine [1]. In addition, it is necessary to design vaccine antigens which induce a potent and broadly neutralizing antibody response against various HIV-1 strains [1], [2]. Plasma of some HIV-1-infected patients contains potent and broadly reactive neutralizing antibodies, and human monoclonal antibodies with broad and potent neutralizing activity have been established [3], [4], [5], [6], [7], [8]. It is believed that understanding how such neutralizing antibodies are elicited in infected patients may provide valuable insights into developing an effective HIV-1 vaccine.

HIV-1 is characterized by extensive genetic heterogeneity and is divided into four groups: M (major), O (outlying), N (new or non-M, non-O) and P (pending). The viruses in group M, which are responsible for the worldwide HIV-1 pandemic, are further classified into many subtypes and circulating recombinant forms (CRFs) [9]. While subtype B of HIV-1 is the predominant subtype in the Americas, Europe and Australia, there is a growing epidemic of non-B subtypes and CRFs in Africa and Asia. CRF01_AE is prevalent throughout Southeast Asia [9] and is responsible for more than 80% of infection cases in Thailand [10].

In this report, as part of studies to reveal the molecular mechanism of how strong humoral immune responses are elicited in HIV-1-infected patients, we performed a comparative study on the neutralizing activity of plasma derived from rapid and slow progressors residing in northern Thailand, using previously established high throughput neutralization tests with CRF01_AE Env-recombinant, luciferase reporter viruses [11], [12].

## Methods

### Ethics statement

This study was conducted with approval from the ethics committee of the Ministry of Public Health of Thailand and with written informed consent from the patients.

### Study participants and sample collection

We studied plasma samples of drug-naive, HIV-1-infected patients who visited the day care center of Lampang Hospital in the early to middle 2000 s, and who were enrolled in a HIV-1 cohort study. Thirty-four plasma samples were derived from slow progressors (CD4 count >100 cells/cm^3^ at the time of enrollment, healthy for at least 8 years without antiretroviral treatment), whereas 33 plasma samples were derived from rapid progressors (CD4 count >100 cells/cm^3^ at the time of enrollment, died with AIDS symptoms within 5 years). Plasma samples were heat-inactivated for 1 hr at 56°C and subjected to neutralization tests.

### Cells

293T cells were maintained in Dulbecco's modified Eagle's medium supplemented with 10% fetal bovine serum (10% FBS-DMEM). U87.CD4.CCR5 and U87.CD4.CXCR4 cells [13] were obtained from Dr. HongKui Deng and Dr. Dan R. Littman through the AIDS Research and Reference Reagent Program (ARRRP), Division of AIDS, NIAID, NIH, and were maintained in 10% FBS-DMEM with puromycin (1 µg/ml) and G418 (300 µg/ml) (complete medium).

### Viral constructs

CRF01_AE-Env (AE-Env)-recombinant, luciferase reporter proviral constructs containing the CRF01_AE *env* genes, 29CC1, 41CC1, 47CC1, 55PL1, 98CC2, 102CC2 and 105PL3, were generated as described previously [12]. The expression vectors for 5 subtype B Env (B-Env), QH0692.42, TRO.11, pWITO4160.33, pREJO4541.67 and SC422661.8 [14], [15], [16], and the vectors for 6 subtype C Env (C-Env), ZM214M.PL15, ZM249MPL1, ZM53M.PB12, ZM109F.PB4, ZM135M.PL10a and CAP210.2.00.E8 [17], were obtained from Drs. Cynthia A. Derdeyn, Feng Gao, Beatrice H. Hahn, Eric Hunter, Ming Li, Yingying Li, Koleka Milsana, David C. Montefiori, Lynn Morris and Jesus F. Salazar-Gonzalez through the ARRRP. Subtype B and C *env* genes were amplified from these expression vectors by polymerase chain reaction and inserted into pNL4-3-derived luciferase reporter viral DNA, pNL-envCT to generate B-Env- and C-Env-recombinant, luciferase reporter proviral constructs, essentially as described [12], [18].

### Preparation of Env-recombinant viruses

293T cells (2×10^5^ cells/2 ml) were seeded onto a collagen-coated 6-well plate (Iwaki, Tokyo, Japan) 24 h prior to transfection. Env-recombinant viruses were prepared by transfecting 293T cells with a proviral construct (2 µg) using FuGENE HD transfection reagent (Roche, Basel, Switzerland). Forty-eight hours after transfection, viral supernatants were cleared by centrifugation for 5 min at 8,000 rpm and stored as aliquots at −85°C. The viral titer was determined by measuring the concentration of HIV-1 Gag p24 antigen in viral supernatants by enzyme-linked immunosorbent assay (ELISA) (HIV-1 p24 ELISA Kit; BioAcademia, Inc., Osaka, Japan).

### Neutralization tests

The neutralization susceptibility of Env-recombinant viruses to plasma samples was examined, essentially as described previously [11]. Briefly, U87.CD4.CXCR4 or U87.CD4.CCR5 cells were incubated with 2-fold serially diluted plasma in 100 µl complete medium for 1 hr at 37°C. U87.CD4.CXCR4 cells were used as target cells for recombinant viruses containing CXCR4-tropic AE-Env, 98CC2 and 107CC2, and dual-tropic AE-Env, 29CC1 and 41CC1. In addition, U87.CD4.CCR5 cells were used as target cells for recombinant viruses containing CCR5-tropic AE-Env, 47CC11, 55PL1, 102CC2 and 105PL3, 2 dual-tropic AE-Env, 5 B-Env and 6 C-Env. The cells were then incubated with viral supernatants (2 ng of p24 antigen) for 48 hrs. Luciferase activity in infected cells was measured using the Steady Glo Luciferase assay kit (Promega) with an LB960 microplate luminometer (Berthold, Bad Wildbad, Germany). The inhibitory effect of the plasma on viral replication was evaluated as a reduction in luciferase activity in infected cells. The reciprocal plasma dilution, at which viral replication was suppressed by 50% (50% inhibitory dilution, ID50), was calculated by the dose-response curve using a standard function of GraphPad Prism 5 software (GraphPad Software, San Diego, CA).

### Statistical analyses

Fisher's exact test was performed to compare the breadth of neutralizing activity in plasma derived from rapid and slow progressors. Briefly, a 2×2 contingency table, consisting of the numbers of plasma/virus combinations in which viral neutralization was observed and total plasma/virus combinations on both groups, was constructed, and the 2-tailed p-value was calculated using QuickCalcs (GraphPad software; http://www.graphpad.com/quickcalcs/). In addition, Student' t-test was performed to compare the potency of neutralizing plasmas derived from rapid and slow progressors, using the standard function of Microsoft Excel (Microsoft Office for Mac 2011; Microsoft, Redmond, WA).

## Results

### Neutralizing activity of plasma derived from rapid progressors

**Figure 1:**
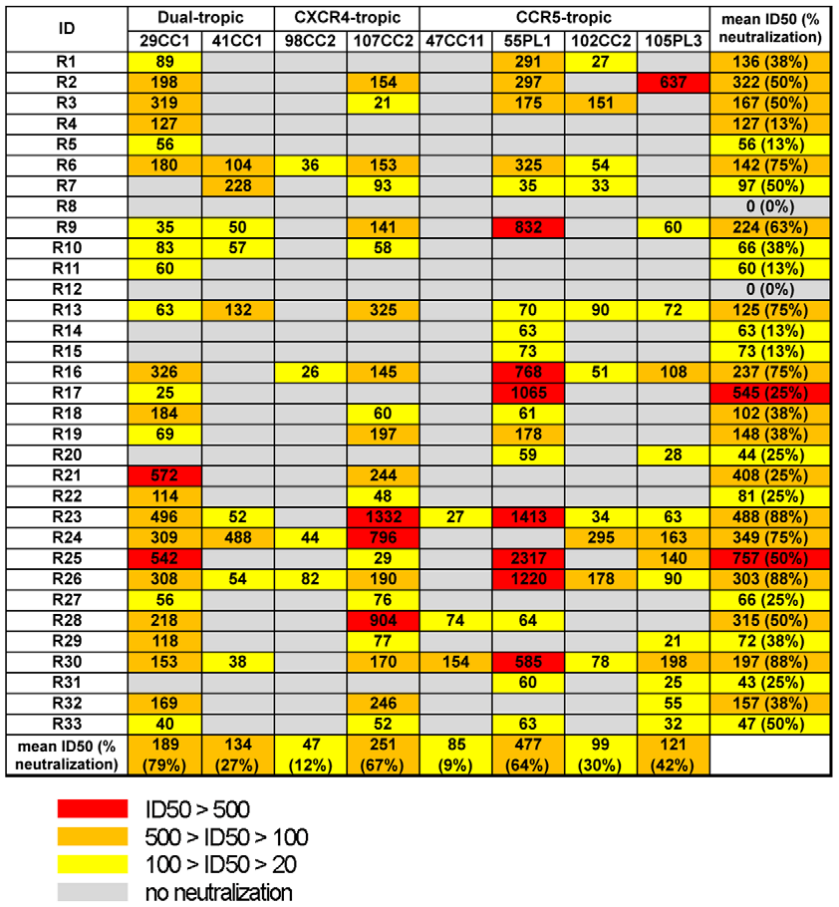
Anti-HIV-1 neutralizing activity of plasma derived from 33 rapid progressors against AE-Env-recombinant viruses. Neutralizing activity of plasma samples against 8 AE-Env-recombinant viruses was evaluated and reciprocal plasma dilution at which viral replication was suppressed by 50% (50% inhibitory dilution, ID50) was calculated, as described in Methods. Data are presented as the means of at least three independent experiments. Plasma IDs and AE-Env-recombinant viruses tested are denoted on the left side and above the panel, respectively. In addition, mean ID50 values and the percentages of virus/plasma combinations (% neutralization) in which viral neutralization was observed among the data sets in horizontal and vertical directions are shown on the right side and bottom of the panel, respectively. ID50 values >500, values 100–500, and values 20–100 are highlighted in red, orange and yellow, respectively. In addition, no neutralization (ID50 values <20) of a recombinant virus is denoted by a gray background.

We evaluated the anti-HIV-1 neutralizing activity of plasma derived from 33 rapid progressors by measuring the inhibitory effect of plasma on a single round replication of previously established AE-Env-recombinant viruses [11], [12]. The 8 AE-Env-recombinant viruses used in this study consisted of the recombinant viruses containing 2 dual-tropic AE-Env, 29CC1 and 41CC1, 2 CXCR4-tropic AE-Env, 98CC2 and 107CC2, and 4 CCR5-tropic AE-Env, 47CC11, 55PL1, 102CC2 and 105PL3 [12]. Plasma samples derived from 33 rapid progressors showed various levels of neutralizing activities against 8 AE-Env-recombinant viruses (Fig. 1). The replication of recombinant viruses containing AE-Env, 29CC1, 55PL1 and 107CC2, was inhibited by many plasma samples, whereas that of recombinant viruses, containing AE-Env, 47CC11 and 98CC2, was inhibited only by 3 and 4 plasma samples, respectively (Fig. 1). The inhibitory effect of plasma on the replication of 2 recombinant viruses containing dual-tropic AE-Env, 29CC1 and 41CC1, in U87.CD4.CCR5 was somewhat, but not significantly higher than that in U87.CD4.CXCR4 cells (data not shown), suggesting that viral entry through the CCR5 molecule is more susceptible to plasma-mediated neutralization than entry through CXCR4. Finally, plasma samples R23 and R30 inhibited the replication of most AE-Env-recombinant viruses tested, but failed to inhibit the replication of a recombinant virus containing AE-Env, 98CC2 (Fig. 1).

### Neutralizing activity of plasma derived from slow progressors

**Figure 2:**
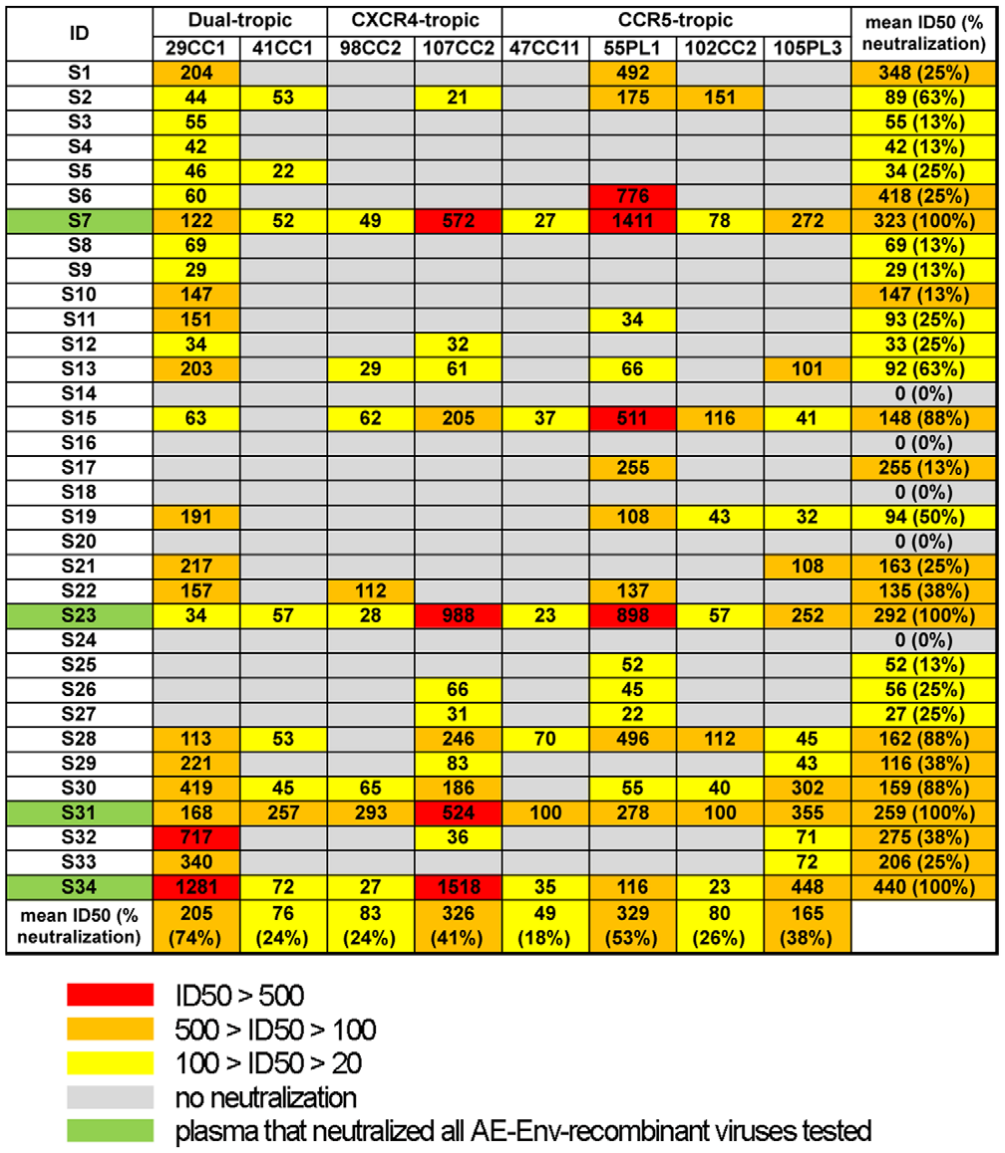
Anti-HIV-1 neutralizing activity of plasma derived from 34 slow progressors against AE-Env-recombinant viruses. Neutralizing activity of plasma samples against 8 AE-Env-recombinant viruses was evaluated as described in the legend to Figure 1. Data are presented as the means of at least three independent experiments. Plasma IDs and AE-Env-recombinant viruses tested are denoted on the left side and above the panel, respectively. In addition, mean ID50 values and the percentages of virus/plasma combinations (% neutralization) in which viral neutralization was observed among the data sets in horizontal and vertical directions are shown on the right side and bottom of the panel, respectively. ID50 values >500, values 100–500 and values 20–100 are highlighted in red, orange and yellow, respectively. No neutralization (ID50 values <20) of a recombinant virus is denoted by a gray background. Plasma samples that neutralized all recombinant viruses tested are highlighted in green.

We next evaluated the neutralizing activity of plasma derived from 34 slow progressors. The plasma samples showed various levels of neutralizing activity against AE-Env-recombinant viruses tested (Fig. 2). Plasma derived from slow progressors inhibited the replication of 8 AE-Env-recombinant viruses with a similar tendency to plasma derived from rapid progressors; however, the replication of 2 recombinant viruses containing AE-Env, 47CC11 and 98CC2 was inhibited more frequently by plasma derived from slow progressors than from rapid progressors (Figs. 1 and 2). In contrast, the replication of a recombinant virus containing AE-Env, 107CC2 was inhibited less frequently by plasma derived from slow progressors than from rapid progressors (Figs. 1 and 2). It is noteworthy that 4 plasma samples, S7, S23, S31 and S34, derived from slow progressors, inhibited the replication of all AE-Env-recombinant viruses tested, whereas no plasma from rapid progressors inhibited the replication of all recombinant viruses (Figs. 1 and 2).

### Comparison of the breadth and potency of anti-HIV-1 neutralizing activity between the plasma derived from rapid and slow progressors

**Figure 3:**
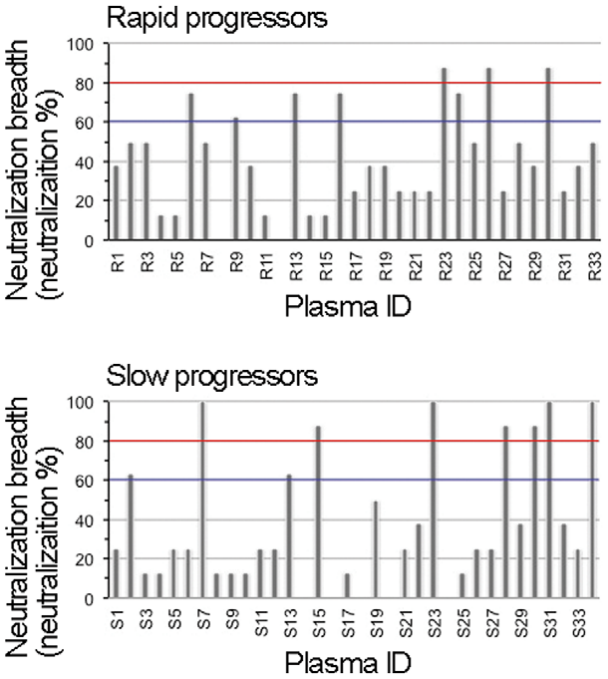
Comparison of the neutralization breadth between plasma derived from rapid and slow progressors. The proportion of AE-Env-recombinant viruses in which replication was inhibited by a plasma sample was calculated and plotted. The levels of plasma-mediated neutralization against 60% and 80% of recombinant viruses tested are highlighted by horizontal blue and red grid lines, respectively. Plasma IDs are denoted below the panels.

We next performed statistical analyses to compare the breadth and potency of neutralizing activity between the plasma derived from rapid and slow progressors. Viral neutralization was observed in 109 virus/plasma combinations among 264 virus/plasma combinations by the plasma derived from rapid progressors (Fig. 1), whereas it was observed in 101 virus/plasma combinations among 277 virus/plasma combinations by the plasma derived from slow progressors (Fig. 2). Fisher's exact test for a 2×2 contingency table revealed no statistical significance (P>0.5) in the breadth of viral neutralization between plasma derived from both groups. In addition, the mean ID50 values of plasma derived from rapid and slow progressors on viral neutralization were 227 and 200, respectively. Student's t-test revealed no statistical significance (P>0.5) in the potency of viral neutralization between plasma derived from both groups; therefore, we concluded that no clear differences were observed overall in the potency and breadth of anti-HIV-1 neutralizing activity in plasma derived from rapid and slow progressors. We next compared the proportion of AE-Env-recombinant viruses neutralized by a plasma sample and evaluated the neutralization breadth of plasma derived from rapid and slow progressors in more detail. The results showed that the replication of more than 60% (5 of 8) of AE-Env-recombinant viruses was inhibited by 24% (8 of 33) and 26% (9 of 34) of plasma derived from rapid and slow progressors, respectively (Fig. 3, bars above blue lines), suggesting no clear difference between the groups. In contrast, the replication of more than 80% (7 of 8) of AE-Env-recombinant viruses was inhibited by 21% (7 of 34) of plasma derived from slow progressors, whereas that was inhibited by 9% (3 of 33) of plasma derived from rapid progressors (Fig. 3, bars above red lines). These results showed that several plasma samples derived from slow progressors neutralized AE-Env-recombinant viruses more frequently than those from rapid progressors.

### Plasma derived from Thai patients possessed CRF01_AE-specific neutralizing activity

We next studied the neutralizing activity of plasma, R23, R30, S7, S23, S31 and S34, which efficiently inhibited the replication of AE-Env-recombinant viruses, against 5 B-Env- and 6 C-Env-recombinant viruses. The results showed that these plasma samples were able to inhibit the replication of less than half of B-Env- and C-Env-recombinant viruses tested.

## Discussion

In this report, we performed a comparative study on the anti-HIV-1 neutralizing activity of plasma samples derived from rapid and slow progressors residing in northern Thailand. Previous reports showed that broadly neutralizing activity against heterologous HIV-1 clinical isolates was more frequently detected in plasma or serum samples derived from long-term non-progressors (LTNP) than from progressors [19], [20], [21], suggesting the involvement of a broadly reactive neutralizing antibody response in regulating disease progression. In contrast, recent reports have shown that broadly neutralizing activity was more frequently detected in serum samples derived from slow and rapid progressors than from LTNP [22], [23]. In addition, no correlation between the neutralization breadth of plasma and disease progression was observed in a recent longitudinal study of a seroincident cohort [24]; therefore, the role of the broadly neutralizing antibody response in HIV-1 disease control is unclear. Nevertheless, the anti-HIV-1 antibody response is a factor involved in regulating viral replication; therefore, it may be important to accumulate knowledge about how a strong humoral immune response is induced in some patients.

No statistically significant differences in the potency and breadth of neutralizing activities were observed overall in plasma derived from two groups of patients in this report (Figs. 1 and 2); however, several plasma samples derived from slow progressors were capable of neutralizing AE-Env-recombinant viruses more frequently than those from rapid progressors (Fig. 3), indicating the possible involvement of the anti-HIV-1 broadly reactive antibody response, at least in part, in controlling disease progression. In addition, broadly neutralizing human monoclonal antibodies, VRC01 and PG9, were recently established from samples derived from a slow progressor and an elite neutralizer, respectively [7], [8], [25], indicating the possible correlation between the induction of potent and broadly neutralizing antibodies and slow disease progression. Broadly neutralizing antibodies are reported to be elicited in the early phase of HIV-1 infection [26]. In addition, such broadly reactive antibodies were elicited in an elite neutralizer within a year after viral infection [27]. Another report showed a correlation between viral load and the broadly neutralizing antibody response, as well as an inverse correlation between CD4 count and such an antibody response, in the early phase of HIV-1 infection [28]. Considering these reports, the induction mechanism of broadly neutralizing antibodies has been elucidated in part, but more information needs to be accumulated. The understanding of broadly neutralizing antibody responses induced by natural HIV-1 infection may provide valuable insights into the design of an effective HIV vaccine antigen using a reverse engineering approach. We studied the epitopes of anti-HIV-1 neutralizing antibodies in selected plasma samples that neutralized AE-Env-recombinant viruses efficiently; however, they were not revealed in this study. We consider that epitope analysis of broadly reactive plasma, as well as evaluation of the immunogenicity of Env gp120 and gp41 molecules derived from viruses isolated from patients with broadly neutralizing plasma, may be important in future studies.

**Figure 4:**
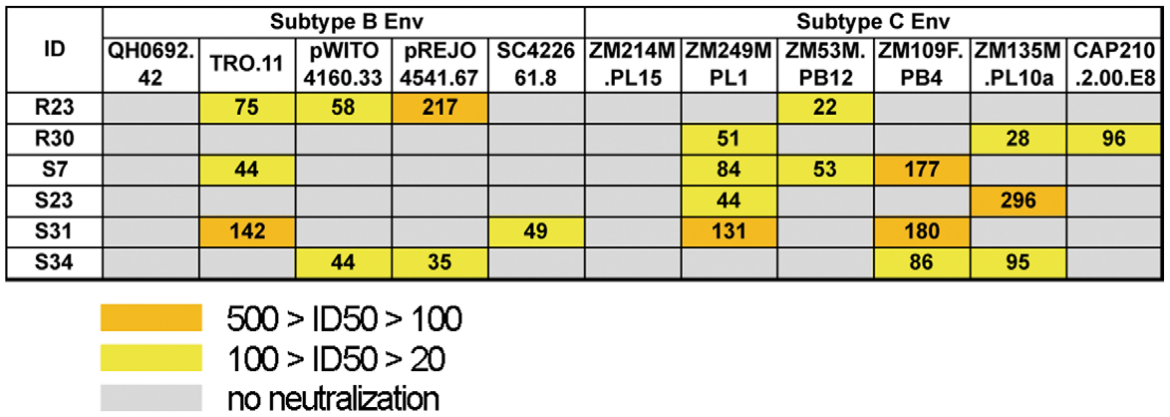
Anti-HIV-1 neutralizing activity of 6 selected plasma samples against B-Env- and C-Env-recombinant viruses. Neutralizing activity of 6 plasma samples against 5 B-Env- and 6 C-Env-recombinant viruses was evaluated as described in the legend to Figure 1. Data are presented as the means of at least three independent experiments. Plasma IDs and Env-recombinant viruses tested are denoted on the left side and above the panel, respectively. ID50 values 100–500 and 20–100 are highlighted in orange and yellow, respectively. No neutralization (ID50 values <20) of a recombinant virus is denoted by a gray background.

The replication of B-Env- and C-Env-recombinant viruses was not efficiently inhibited by selected plasma that showed broadly neutralizing activity against AE-Env-recombinant viruses (Fig. 4). These results suggest that the antigenicity of Env gp120 and gp41 differs among CRF01_AE, subtype B and C viruses. Our results were consistent with the results described in a previous report that serum samples derived from subtype B and E (CRF01_AE) -infected Thai individuals showed subtype-specific neutralizing activity [29]. Env gp120 and gp41 are the most variable HIV-1 proteins, with typical intersubtype and intrasubtype differences reaching 35% and 20%, respectively [30]. In addition, structural differences between subtype B and C Env molecules have recently been reported [31]. Moreover, our recent observations suggested that different Env regions were affected by host immune pressure between CRF01_AE and subtype B viruses [32]. Taken together with the results in this report, we believe that it is important to take into account the antigenic and immunogenic diversity among different subtypes and CRFs of HIV-1 in developing HIV-1 vaccine antigens to elicit a broadly neutralizing antibody response.
